# Italian translation, cultural adaptation, and pilot testing of a questionnaire to assess family burden in inherited ichthyoses

**DOI:** 10.1186/s13052-019-0618-x

**Published:** 2019-02-19

**Authors:** May El Hachem, Damiano Abeni, Andrea Diociaiuti, Roberta Rotunno, Francesco Gesualdo, Giovanna Zambruno, Christine Bodermer

**Affiliations:** 1 Dermatology Unit, Bambino Gesù Children’s Hospital, IRCCS, Rome, Italy; 2 Clinical Epidemiology Unit, IDI-IRCCS, Rome, Italy; 3 Multifactorial and Complex Disease Research Area, Bambino Gesù Children’s Hospital IRCCS, Rome, Italy; 4 Department of Dermatology, Necker-Enfants Malades Hospital, Centre de Référence National pour les Maladies Génétiques à Expression Cutanée (MAGEC), APHP, Paris, France

**Keywords:** Inherited ichthyosis, Autosomal recessive congenital ichthyosis (ARCI), Quality of life, Family burden, Dermatology

## Abstract

**Background:**

Inherited ichthyoses are rare disorders characterized by generalized skin scaling. Among them, autosomal recessive congenital ichthyoses (ARCI) form a major subgroup presenting lifelong and severely disabling cutaneous and extracutaneous features and symptoms for which no curative treatment is available. Management relies on daily time-consuming and distressing topical medications. Disease manifestations, symptoms, and daily care affect not only the patient self-perception, but also different dimensions of patient and family life. To date, there is only a French validated ichthyosis-specific questionnaire, “Family Burden in Ichthyosis” (FBI), for the evaluation of family disease burden. It addresses economical aspects, daily life, familial and personal relationships, work, and psychological impact. The aim of our study was to develop an Italian translation of the French FBI questionnaire and to pilot-test it in ARCI patients.

**Methods:**

The guidelines for cross-cultural adaptation of health-related quality of life measures were followed. Specifically, two independent forward translations were produced, followed by a reconciliation step by a multidisciplinary expert committee and back-translation. Revision of the original text and all translations was performed by the expert committee leading to a final version, which was pilot-tested by cognitive debriefing on 10 caregivers whose comments were evaluated by the committee.

**Results:**

The translation and reconciliation process led to minor changes in five items in order to clarify the questions in relation to the possible answers or to obtain semantic/idiomatic/cultural equivalence of the Italian version with the French one. The cognitive debriefing process resulted into further minor wording modifications in four items to describe more precisely the disease impact according to parents’ comments. The FBI developer approved the final Italian FBI version.

**Conclusions:**

The Italian version of the FBI generated in the present study is a useful instrument to measure the impact of ichthyosis on family daily life, education and working activities, psychological implications, and the disease economic load. The questionnaire will be further validated through a multicenter Italian study on burden of ARCI. A validated Italian questionnaire is a valuable tool for future clinical trials. In addition, it can be used to rapidly identify family distressing situations, which require attention and prompt intervention.

## Background

**Fig. 1:**
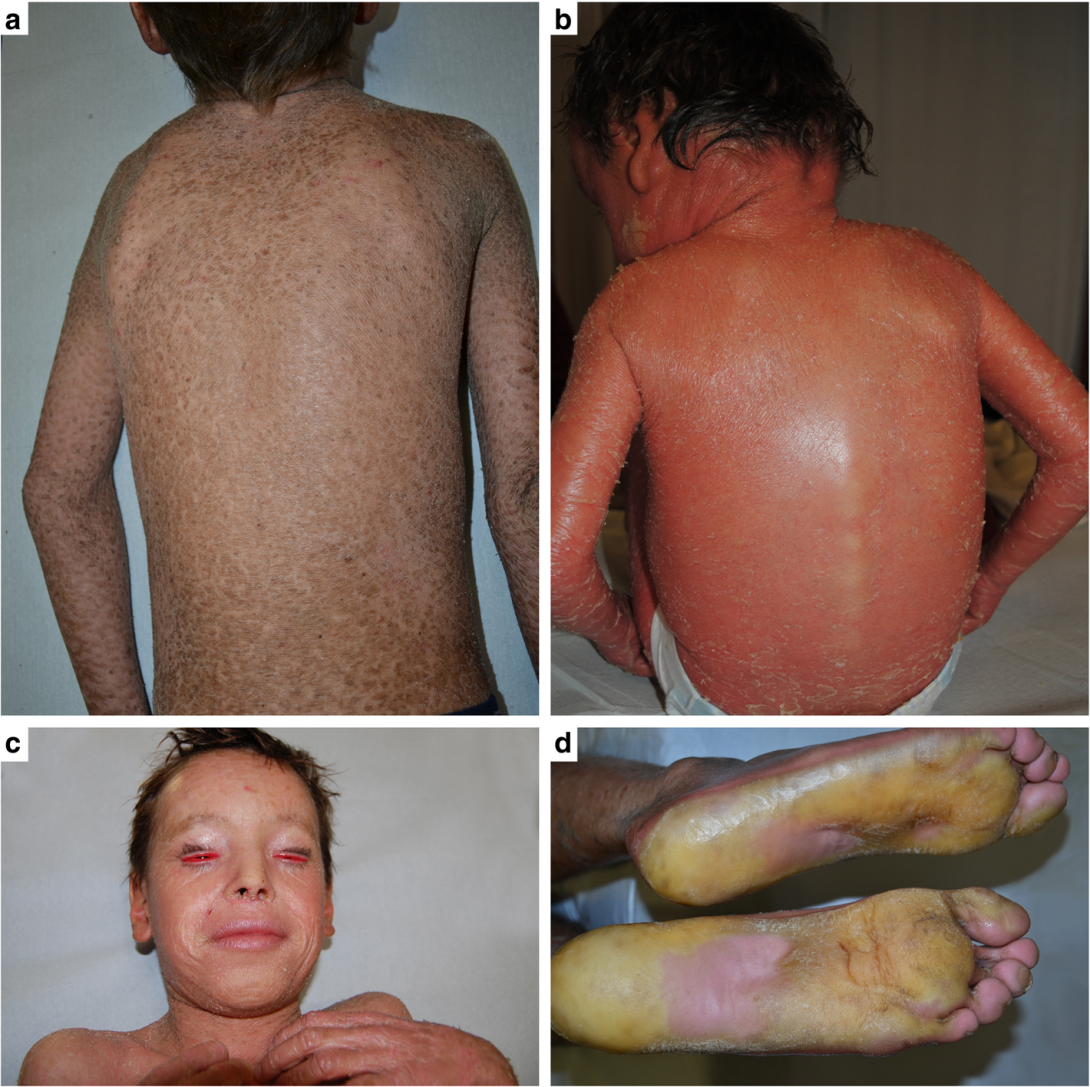
– Inherited ichthyosis clinical features. 9-year-old boy affected by autosomal recessive congenital ichthyosis (ARCI) showing generalized skin involvement with dark, large, and adherent scales (**a**), while a 3-year-old child also affected with ARCI presents diffuse skin erythema (erythroderma), in addition to whitish scales (**b**); the child shown in (**a**) also has bilateral ectropion (upper and lower lid eversion), a common and severe disease complication (**c**); plantar keratoderma in a 17-year-old ARCI patient (**d**)

**Fig. 2:**
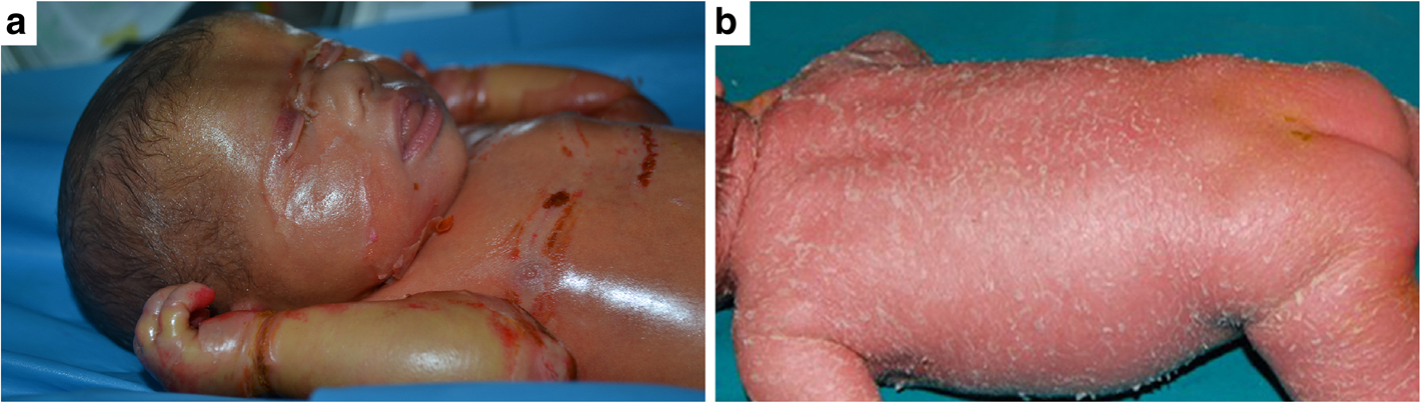
- Autosomal recessive congenital ichthyosis at birth. The more common presentation is a parchment-like membrane, the so-called collodion membrane, covering the newborn, with fissures on the chest, abdomen, and upper limbs and constrictive bands on the fingers (**a**). Less frequently, the newborn presents a generalized erythroderma with fine-whitish scaling (ichthyosiform erythroderma) (**b**)

Inherited ichthyoses comprise a clinically and genetically heterogeneous group of disorders, characterized by generalized skin scaling and hyperkeratosis often associated with erythema [1]. With the exception of ichthyosis vulgaris, all ichthyosis forms are rare diseases with onset in most cases at birth. Whole body skin scaling and, in a number of patients, erythema (Fig. 1a, b) completely alter the patient’s physical appearance and affect his/her self-perception. Additional disease features include ectropion (lid eversion) (Fig. 1c), hearing impairment due to ear canal scaling, foul-smelling skin, limited function due to joint contractures and palmoplantar keratoderma (Fig. 1d), alopecia, hypohidrosis with thermodysregulation, frequent infections, severe pruritus, and skin pain. All these strongly affect quality of life (QoL) of the patients and their families [2–5]. Autosomal recessive congenital ichthyoses (ARCI) represent a major subgroup of ichthyoses, with onset at birth: the newborn is either entirely wrapped by a parchment-like membrane, the so-called collodion membrane or, less commonly, shows generalized erythroderma with scaling (ichthyosiform erythroderma) (Fig. 2) [1]. Both presentations usually require hospitalization in a Neonatal Intensive Care Unit due to highly defective skin barrier function [6, 7]. When the collodion membrane detaches, the baby gradually develops the above-mentioned disabling clinical features. As no curative treatment is available, patient care relies on daily bathing, followed by whole-body application of topical emollients, keratolytics, and retinoids [6]. Addition of oral retinoids is frequently required in ARCI [6]. Patient care is thus highly time-consuming and distressing for both patients and caregivers [2, 3]. The impact of disease manifestations and patient care on QoL has been at first evaluated by specialty-specific questionnaires, in particular the Dermatology Life Quality Index [2–5, 8]. Only recently, two disease-specific instruments have been developed in France to globally evaluate family disease burden including psychological, social, and economic aspects, and consequences of physical features [9, 10]. However, only one questionnaire, named “Family Burden in Ichthyosis (FBI)”, has been validated in patients affected with ARCI [9].

The aim of our study was to develop an Italian translation of the French original version of the FBI questionnaire and to pilot test it.

## Methods

### Original questionnaire

The FBI is a self-administered questionnaire, which comprises 25 items addressing five domains: economical aspects, daily life, familial and personal relationships, work, and psychological impact [9]. Answers are given on a 4-point Likert scale: definitely yes, sometimes, definitely not, I do not know. Higher scores indicate a greater family burden of ichthyosis. The FBI has been shown to correlate with the mental scale of short form-12 (SF-12), and its five dimensions with the disease severity score.

### Translation

The Ethical Committee of the Bambino Gesù Children’s Hospital (OPBG) approved the study of the Italian translation, cultural adaptation, and pilot testing of the FBI questionnaire as a part of a cognitive multicenter study on disease burden in ARCI ichthyoses. The guidelines for cross-cultural adaptation of health-related QoL measures were followed [11]. Specifically, a forward translation was produced independently by two native Italian speakers and then underwent a reconciliation step by an expert committee, according to the following criteria: the translation should reflect the original French text, and Italian culture must be taken into account in choosing the words and constructing the sentences. The draft Italian text was then back-translated by two French mother tongue speakers and sent to the Authors for checking. Then, the expert committee further revised the original text and all translations, evaluating equivalence between the source and the translated questionnaires in the areas of semantic, idiomatic, experiential and conceptual equivalence. The pre-pilot testing version was submitted to the developers of the FBI for approval together with an interim report.

### Pilot testing

Following approval by the FBI developers, pilot testing of the Italian translated version of the FBI was performed by cognitive debriefing on 10 caregivers who gave written informed consent. The participants were recruited from families, with at least one child affected with ARCI, attending the Reference Centre for Rare Skin Diseases of OPBG. A dermatologist contacted the parents, explained the aims of the project, and enrolled the parents who gave their consent. The parents completed the questionnaire on their own, after being instructed to mark questions they found unclear or difficult to understand or to answer. After the questionnaire was completed the participants were interviewed by the dermatologist, who enquired about the reasons why some questions were problematic, and also asked how they would have rephrased in their own words the questions that they identified as difficult or unclear. The interviewer kept notes of the family comments on a standardized form.

### Data analysis

The expert committee reviewed the results of all interviews, prepared a summary of participants’ comments and made decisions on all questions identified as problematic. The final version of the questionnaire was submitted to the Authors of the French FBI for final approval.

## Results

The forward translation was performed in parallel by an experienced dermatologist (GZ) and a professional translator. The translations were evaluated for reconciliation by the expert committee, which comprised epidemiologists (FG, DA), dermatologists (AD, GZ, MEH, RR), and language professionals. In 20 items, there was full agreement between translators and correspondence with the original French version.

The wording of the remaining five items was slightly modified in order to make clearer the questions in relation to the different answer options (3 items) or to obtain semantic/idiomatic/cultural equivalence of the Italian version with the French one (2 items) (Table 1).

**Table 1:** Concerns and comments from expert committee explaining changes

Question N.	Concerns	Discussion and final choice
13	The adverb “often” does not seem to fit well with the answer options, in particular with “sometimes”	Considering the range of possible answers it was decided to omit the word “often” from the question
16	The adverb “completely” does not seem to fit well with the answer options	This adverb has been removed from the question
18	The Italian translation of the expression “to the smell caused by my child’s skin disease” could be confusing	A more direct wording: “to the smelling of the skin of my child” has been adopted in Italian without modifying the sentence meaning
19	The verb “to babysit” does not have an Italian translation	Considering that the word *babysitter* is currently used in Italian, it has been adopted in the Italian version
20	The expression “a lot of difficulties” implies that only major difficulties will be considered in the answers	The word “a lot” has been removed because the answers already allow to grade the entity and frequency of the difficulties if encountered

Following reconciliation, one French mother tongue translator and a French mother tongue dermatologist (MEH) back translated the Italian text. There was complete agreement between the two translators. The Authors of the FBI approved the initial back translation. The committee then revised the original questionnaire and all translations, and evaluated equivalence between the source and the translated questionnaires. The pre-pilot version was approved by the FBI Authors.

Cognitive debriefing performed on 10 parents of five children affected with ARCI did not reveal any particular problem in the comprehension of the 25 questionnaire items. However, the committee slightly modified single words in four items to more appropriately describe disease impact according to parents’ comments (Table 2). The validated Italian text was forwarded again to the developer for final approval (Table 3).

**Table 2:** Concerns and comments from parents explaining changes

Question N.	Comments	Suggested wording	Final choice
16	The expression “disrupted my life” sounded extreme to all parents	The parents suggested alternative words: “affects my life” (6 parents), and “modifies my life” (4)	“Affects” has been adopted in the final version
23 and 24	The expression “I don’t feel well” did not reflect the real feeling	The suggested alternatives were: “I am anxious” (4 parents), “I am distressed” (2), “I am worried” (2), and “I am nervous” (2)	“I feel anxious” has been chosen for the final version
25	The parents considered excessive the expression “extremely tiring”	The proposals were: “tiring” without any adjective (4 parents), and “very tiring” (6)	To conciliate the need for correct translation and the patient request, the chosen expression was “very tiring”

**Table 3:** Italian version of the family burden in inherited ichthyosis questionnaire

Per ognuna delle seguenti affermazioni, per cortesia, risponda nella maniera più spontanea possibile pensando alla Sua situazione negli ultimi 7 giorni. Per favore indichi una sola risposta					
		Certamente sì	Qualche volta	Assolutamente no	Non so
1	La malattia della pelle di mio/a figlio/a ci ha spinto a pensare di trasferirci				
2	La malattia della pelle di mio/a figlio/a mi ha portato a pensare di lasciare il mio lavoro				
3	La malattia della pelle di mio/a figlio/a incide sul mio sonno				
4	La malattia della pelle di mio/a figlio/a influisce sulla nostra vita familiare				
5	Penso alla malattia della pelle di mio/a figlio/a tutto il giorno				
6	La malattia della pelle di mio/a figlio/a ci impedisce di andare in vacanza				
7	Mio/a figlio/a ha bisogno di più attenzione degli altri bambini a causa della sua malattia della pelle				
8	La malattia della pelle di mio/a figlio/a ci ha costretti a rimettere in discussione i nostri progetti per il futuro				
9	La malattia della pelle di mio/a figlio/a ci fa trascurare gli altri figli				
10	Non riesco ad andare a trovare la mia famiglia a causa della malattia della pelle di mio/a figlio/a				
11	La mia famiglia non viene a trovarci a causa della malattia della pelle di mio/a figlio/a				
12	La malattia della pelle di mio/a figlio/a ci crea dei problemi di coppia				
13	Le visite per la malattia della pelle di mio/a figlio/a mi danno spesso un senso di frustrazione				
14	Le reazioni della gente di fronte alla malattia della pelle di mio/a figlio/a sono difficili da accettare				
15	Mi sento in colpa a causa della malattia della pelle di mio/a figlio/a				
16	La malattia della pelle di mio/a figlio/a ha profondamente condizionato la mia vita				
17	Faccio fatica ad accettare la malattia della pelle di mio/a figlio/a				
18	Faccio fatica ad abituarmi all’odore della pelle di mio/a figlio/a				
19	Ho grandi difficoltà a trovare una babysitter per mio/a figlio/a a causa della sua malattia della pelle				
20	Mio/a figlio/a ha difficoltà a scuola a causa della sua malattia della pelle				
21	Ho paura per il futuro di mio/a figlio/a a causa della sua malattia della pelle				
22	Le cure quotidiane iniziano a pesarmi				
23	La sera prima di andare in ospedale, sono in ansia				
24	Il giorno dopo essere andato/a in ospedale, sono in ansia				
25	Le cure necessarie a mio/a figlio/a sono molto stancanti				

## Discussion

In the field of skin diseases, only eight validated questionnaires for the measure of the family burden of specific diseases exist [12]. Two of these address rare skin diseases of pediatric interest: epidermolysis bullosa (epidermolysis bullosa burden of disease) [13] and the FBI for ichthyosis [9]. Since only one specialty-specific questionnaire has been validated in Italian, i.e., the Family Dermatology Life Quality Index (FDLQI) [14, 15], and we are planning a multicenter study on ichthyosis burden in our country, we decided to translate and validate the disease-specific FBI. This study will be carried out within the framework of the European Reference Network (ERN) for rare and undiagnosed skin diseases, to which most of the participating centers belong.

Indeed, rare and chronic diseases pose a major burden on patient and family QoL. Therefore, questionnaires designed to measure the impact of diseases such as ichthyosis on family daily life, education and working activities, the disease economic load, and its psychological and social effects are valuable instruments [13–16].

The generation of the Italian version of the FBI did not pose major cultural adaptation issues also due to the cultural similarities between Italy and France. In addition, both National Health Systems have designated a network of reference centers for rare disorders and cover, in part, the costs for topical and systemic drugs, and other products and devices necessary for patient care.

The main limit of our study has been the cognitive debriefing performed on a small sample of ten caregivers in a single center. However, the validation process has strictly followed the guidelines for cross-cultural adaptation of health-related QoL measures, and an excellent agreement between both the researchers/translators and the caregivers was registered. Nevertheless, the Italian FBI will undergo further validation, including the verification of its psychometric properties, during the planned multicenter Italian study on ichthyosis burden.

The French FBI has been validated by 42 families showing a strong correlation of all questionnaire dimensions, in particular “daily life”, “economic” and “familial/personal relationships”, with disease severity [9]. Our planned multicenter Italian study on ichthyosis burden will allow to evaluate if these findings are also valid for the Italian population.

## Conclusions

The availability of a validated Italian questionnaire can represent meaningful outcome measure in future clinical studies, including controlled trials. Finally, this instrument can be used in the routine clinical practice of reference centers to rapidly and more precisely identify specific psychological critical situations in the families of children with ARCI, which require great attention and prompt intervention and treatment.

## Abbreviations

ARCI: Autosomal recessive congenital ichthyosis
FBI: Family burden in inherited ichthyosis
OPBG: Bambino Gesù Children’s Hospital
QoL: Quality of life
