# Correction to: Italian translation, cultural adaptation, and pilot testing of a questionnaire to assess family burden in inherited ichthyoses

**DOI:** 10.1186/s13052-020-0791-y

**Published:** 2020-03-11

**Authors:** May El Hachem, Damiano Abeni, Andrea Diociaiuti, Roberta Rotunno, Francesco Gesualdo, Giovanna Zambruno, Christine Bodemer

**Affiliations:** 1 Dermatology Unit, Bambino Gesù Children’s Hospital, IRCCS, Rome, Italy; 2 Clinical Epidemiology Unit, IDI-IRCCS, Rome, Italy; 3 Multifactorial and Complex Disease Research Area, Bambino Gesù Children’s Hospital IRCCS, Rome, Italy; 4 Department of Dermatology, Necker-Enfants Malades Hospital, Centre de Référence National pour les Maladies Génétiques à Expression Cutanée (MAGEC), APHP, Paris, France

**Correction to: Ital J Pediatr (2019) 45:26**

**http://doi.org/10.1186/s13052-019-0618-x**

The original article [1] contains a misspelling of co-author, Christine Bodemer’s name which is presented correctly in this Correction article.
